# Geographical and Ethnic Distribution of the HBV C/D Recombinant on the Qinghai-Tibet Plateau

**DOI:** 10.1371/journal.pone.0018708

**Published:** 2011-04-11

**Authors:** Bin Zhou, Lei Xiao, Zhanhui Wang, Ellen T. Chang, Jinjun Chen, Jinlin Hou

**Affiliations:** 1 Institute of Hepatology and Key Lab for Organ Failure Research, Nanfang Hospital, Southern Medical University, Guangzhou, Guangdong, China; 2 Department of Severe Hepatopathy, the Eighth People's Hospital of Guangzhou, Guangzhou, Guangdong, China; 3 Asian Liver Center, Department of Surgery, Stanford University School of Medicine, Stanford, California, United States of America; University of Pretoria/NHLS TAD, South Africa

## Abstract

Two forms of hepatitis B virus (HBV) C/D recombinant have been identified in western China, but little is known about their geographical and ethnic distributions, and particularly the clinical significance and specific mutations in the pre-core region. To address these questions, a total of 624 chronic HBV carriers from four ethnic populations representing five provinces in western China were enrolled in this study. Genotypes were firstly determined by restriction fragment length polymorphism, and then confirmed by full or partial genome nucleotide sequencing. The distribution of HBV genotypes was as follows: HBV/B: 40 (6.4%); HBV/C: 221 (35.4%); HBV/D: 39 (6.3%); HBV/CD: 324 (51.9%). In the 324 HBV C/D recombinant infections, 244 (75.3%) were infected with the “CD1” and 80 (24.7%) were infected with the “CD2.” The distribution of HBV genotypes exhibited distinct patterns in different regions and ethnic populations. Geographically, the C/D recombinant was the most prevalent HBV strain on the Qinghai-Tibet Plateau. Ethnically, the C/D recombinant had a higher prevalence in Tibetan patients than in other populations. Clinically, patients with HBV/CD1 showed significantly lower levels of serum total bilirubin than patients with HBV/C2. The prevalence of HBeAg was comparable between patients with HBV/CD1 and HBV/C2 (63.3% vs 50.0%, *P* = 0.118) whether patients were taken together or stratified by age into three groups (65.6% vs 58.8% in <30 years, *P* = 0.758; 61.9% vs 48.0% in 30–50 years, *P* = 0.244; 64.3% vs 33.3%, *P* = 0.336). Virologically HBV/CD1 had a significantly lower frequency of G1896A than HBV/C2. In conclusion, the HBV C/D recombinant is restricted to the Qinghai-Tibet Plateau in western China and is found predominantly in Tibetans. The predominance of the premature pre-core stop mutation G1896A in patients with the HBV C/D recombinant may account for the higher prevalence of HBeAg in these patients.

## Introduction

Chronic hepatitis B virus (HBV) infection is a serious global health problem and an important cause of morbidity and mortality in endemic areas such as Africa, South-East Asia and China, where most infections are acquired at birth or during childhood. Eight HBV genotypes (A to H) have been identified according to a divergence of more than 8% in the entire nucleotide sequence, and two additional genotypes I and J were tentatively proposed more recently [1], [2], [3]. Subgenotypes have been described within these HBV genotypes based on a divergence in the complete nucleotide sequence greater than 4% and less than 8% [4]. Distinctive geographical and ethnic distributions of HBV genotypes and subgenotypes have also been observed.

An increasing number of HBV intergenotype recombinants have been identified, suggesting that DNA recombination is a relatively frequent event in HBV infection. Recombinant forms B2–B5 formed by recombination between HBV genotype B and C, are the predominant HBV genotype B strains that prevail in South-East Asia [5], [6]. Recombination between genotype A and D was identified in Italy, South Africa and India [7], [8], [9]. Other A/C, A/E and A/G hybrids have also been described [10], [11], [12], [13]. Two studies comprehensively analyzed all available full-length HBV genomes in GenBank/EMBL/DDBJ and reported the frequent occurrence of recombination event between two or more HBV strains [14], [15].

Two forms of recombination between genotype C and D were identified in western China [16], [17]. The first recombinant has genotype D pre-S2/S sequence from nt 10-799 (‘CD1’) while the second has a larger segment extending through the pre-S2/S region to the X gene (nt 10-1499, ‘CD2’). Both recombinants belong to the ayw2 serotype. The D∶C ratio in the 2 recombinants is 25∶75 and 46∶54, respectively. To date the HBV C/D recombinants have only been identified in western China with the exception of one case reported from a Japanese patient [18]. However, the geographical and ethnic distributions of the HBV C/D recombinant, as well as the clinical significance and specific mutations, have not yet been fully studied. Understanding these questions could potentially reveal the origin of the HBV C/D recombinant and benefit the clinical practice in this area. The study determined the prevalence of HBV C/D recombinants in five geographically and ethnically diverse provinces in western China.

## Methods

### Patients

A total of 624 chronic HBV carriers were enrolled from four ethnic Chinese populations including the majority Han, and the other 3 native aboriginal minority populations (Tibetan, Hui and Uygur), representing five regions (Tibet, n = 166; Qinghai, n = 219; Gansu, n = 156; Xinjiang, n = 39; and Ningxia, n = 44) in western China. All patients were seronegative for hepatitis C and hepatitis D viruses. Serum samples from each subject were kept at −30°C until analysis. Written informed consent was obtained from 236 of the participants, and verbal informed consent was obtained from 388 participants. Both the study and the verbal consent were approved by Ethics Committee of Nanfang Hospital.

### HBV genotyping

HBV DNA was extracted from 100 µl serum by using either the QIAamp DNA Blood Mini Kit (Qiagen) or the HBV DNA extraction kit (Huayin Inc., Guangzhou, China). HBV Genotypes were determined firstly by restriction fragment length polymorphism (RFLP), and then by sequencing. The RFLP analysis was performed as described previously [19], with a slight modification. The HBV S region was amplified with primers BS1 (5′-CCTGCTGGTGGCTCCAGTTC-3′, 56–75) and P29 (5′-ATACCCAAAGACAAAAGAAAA-3′, 827–807) for the first round of PCR amplification and primers YS1 (5′-GCGGGGTTTTTCTTGTTGAC-3′, 203–222) and YS2 (5′-GGGACTCAAGATGTTGTACAG-3′, 787–767) for the second round. PCR products were digested separately with restriction endonucleases *Bsrs*I and *Sty*I for genotypes B and C respectively.

PCR products that failed to cut by *Bsrs*I and *Sty*I were cut separately with *Mbo*I and *Pst*I, and then analyzed by electrophoresis on 2% agarose gel stained with ethidium bromide. Because the C/D recombinant has a *Pst*I restriction site at nt 518 in the S gene whereas the site was absent in genotype D. Samples with both *Mbo*I- and *Pst*I-specific digestion were considered as the HBV C/D recombinant, whereas samples with only *Mbo*I digestion were considered as genotype D.

All samples were then also sequenced either partially or completely to confirm the accuracy of the RFLP genotype classification and to distinguish the two types of C/D recombinant. Samples characterized as genotype B and C by RFLP were amplified with primers BS1 and Pol2 (5′-CGGGCAACGGGGTAAAGGTTC-3′, 1157–1137), and if necessary, primers BS1 and P29 were used for semi-nested PCR. The PCR products were analyzed by electrophoresis on 1.5% agarose gel stained with ethidium bromide, and were sequenced using primer BS1 with an ABI 3730 automated DNA sequencer (Applied Biosystems). Genotypes were determined by phylogenetic analyses on the S gene.

Partial and full genome sequence data from samples characterized as HBV/D or C/D recombinant based on RFLP analysis were amplified with primers P1 and P2 as described previously [20], and followed by a semi-nested PCR with primers PreS2 (5′-GGGTCACCATATTCTTGGG-3′, 2814–2832) and P2 to amplify the preS/S plus X gene. All PCR products were sequenced with primer Pol2, and 102 were further sequenced with primer PreS2 or Pol10 (5′-GGTCTTTTGGGCTTTGCTGC-3′, 1002–1022). Additionally, the full-length HBV-DNA of 11 HBV/C, 6 HBV/D, 10 HBV/CD1 and 9 HBV/CD2 were amplified with primers P1 and P2, and were sequenced with an ABI 3730 automated DNA sequencer (Applied Biosystems). Genotypes were determined by phylogenetic analyses based on the sequenced partial or full genome sequences.

### Phylogenetic analysis

The raw sequence data were assembled and analyzed using DNA sequence analysis software (Lasergene software suite V6.0, DNASTAR). The full-length or partial HBV DNA sequences were aligned using CLUSTAL W software (version 1.83; DDBJ) along with HBV A-G genotype reference sequences retrieved from GenBank/EMBL/DDBJ. Genetic distances were estimated by Kimura's 2-parameter method, and phylogenetic trees were constructed by the neighbor-joining method using MEGA software V4 [21]. Bootstrap resampling and reconstruction with 1000 replicates were carried out to confirm the reliability of the phylogenetic trees. Intergenotypic recombination of the 19 full genomes HBV C/D recombinants were searched for with software SimPlot V3.5.1 [22].

### Clinical and virological characteristics of the C/D recombinant

We have shown that the C/D recombinant in western China was derived from the recombination between subgenotype C2 and genotype D, and HBV/C2 and HBV/CD are the most prevalent HBV strains in this area [23]. To investigate the clinical and virological differences between HBV/C2 and HBV/CD1, available clinical data was examined from 109 individuals with CD1 infection and 48 individuals with subgenotype C2 infection from Qinghai province. They were matched for the mean age and sex. All the patients were chronic HBV carriers and were antiviral treatment naive. The pre-core plus core region was amplified and sequenced as described previously [23] and examined for specific mutations.

### Statistical analyses

All data were analyzed by using the statistical package SPSS (version 12.0; SPSS, Inc., Chicago, IL). Chi-square, Fisher's exact, and Student's *t* tests were used as appropriate. A *P* value of <0.05 was considered statistically significant.

### Nucleotide Sequence Accession Numbers

The full-genome HBV nucleotide sequences reported in this article have been submitted to GenBank (HM750131-HM750156, JF491447-JF491456). The subgenomic sequences can be obtained from the authors upon request.

## Results

### Geographical and ethnic distribution of the HBV C/D recombinant

A total of 647 HBsAg positive serum samples were collected from the five provinces. Of which 624 were S gene PCR positive and could be analyzed further. When we performed RFLP using digestion with *Bsrs*I, *Sty*I, *Mbo*I and *Pst*I on 624 samples, 611 could be genotyped by RFLP (B = 40, C = 208, D = 44, CD = 319) and 13 failed to be cut by these endonucleases. All samples were then sequenced to confirm the accuracy of the genotype classification by RFLP. Five samples determined to be genotype D by RFLP were subsequently shown to be C/D recombinant by direct sequencing. The thirteen samples that could not be cut were shown to be genotype C by sequencing. So the distribution of HBV genotypes was as follows: HBV/B: 40 (6.4%); HBV/C: 221 (35.4%); HBV/D: 39 (6.3%); HBV/CD: 324 (51.9%). HBV genotypes B, C, D and C/D recombinant partitioned differently according to geographical regions and ethnic populations; no other genotypes were identified in this study. Based on sequencing and phylogenetic analysis, all genotype B isolates belonged to HBV/B2 subgenotype; 3 genotype C isolates belonged to HBV/C1 and the others belonged to HBV/C2. Among the 324 C/D recombinant isolates, two types of C/D recombinant were identified: 244 belonged to ‘CD1’ and 80 belonged to ‘CD2’.

HBV/CD1 was found in all five provinces (Table 1), with a much higher prevalence than HBV/CD2 in Qinghai, Gansu and Ningxia provinces, whereas in Tibet, the prevalence of ‘CD2’ strain was slightly higher than that of ‘CD1’ strain. HBV/D was also found in all five provinces, but with a very low prevalence, except in Xinjiang, where almost half of the native aboriginal Uygur patients were infected with genotype D and few were infected with the C/D recombinant.

**Table 1:** Distribution of HBV genotypes by geographical region and ethnicity in western China.

Region	Ethnicity	Sex(M/F)	Age (years)*	Genotypes (n)				
				B	C	D	CD1	CD2
Tibet(n = 166)	Han (n = 35)	27/8	15–54 (28.9±10.1)	19 (54.3%)	12 (34.3%)	0 (0.0%)	3 (8.6%)	1 (2.9%)
	Tibetan (n = 131)	64/67	3–54 (23.1±8.2)	1 (0.8%)	9 (6.9%)	3 (2.3%)	54 (41.2%)	64 (48.9%)
Qinghai(n = 219)	Han (n = 66)	37/29	14–84 (36.4±14.1)	2 (3.0%)	33 (50.0%)	4 (6.1%)	25 (37.9%)	2 (3.0%)
	Tibetan (n = 153)	90/63	2–75 (32.8±13.9)	2 (1.3%)	25 (16.3%)	3 (2.0%)	113 (73.9%)	10 (6.5%)
Gansu(n = 156)	Han (n = 125)	90/35	5–70 (29.8±12.0)	13 (10.4%)	93 (74.4%)	4 (3.2%)	13 (10.4%)	2 (1.6%)
	Hui (n = 31)	25/6	2–63 (31.9±18.8)	0 (0.0%)	10 (32.3%)	1 (3.2%)	20 (64.5%)	0 (0.0%)
Ningxia	Hui (n = 44)	30/14	11–63 (37.4±14.1)	1 (2.3%)	23 (52.3%)	5 (11.4%)	15 (34.1%)	0 (0.0%)
Xinjiang	Uygur (n = 39)	28/11	7–60 (27.8±12.0)	2 (5.1%)	16 (41.0%)	19 (48.7%)	1 (2.6%)	1 (2.6%)
Total (n = 624)		391/233	2–90 (32.3±14.4)	40 (6.4%)	221 (35.4%)	39 (6.3%)	244 (39.1%)	80 (12.8%)

*Data are given as age range (means ± standard deviations).

**Figure 1:**
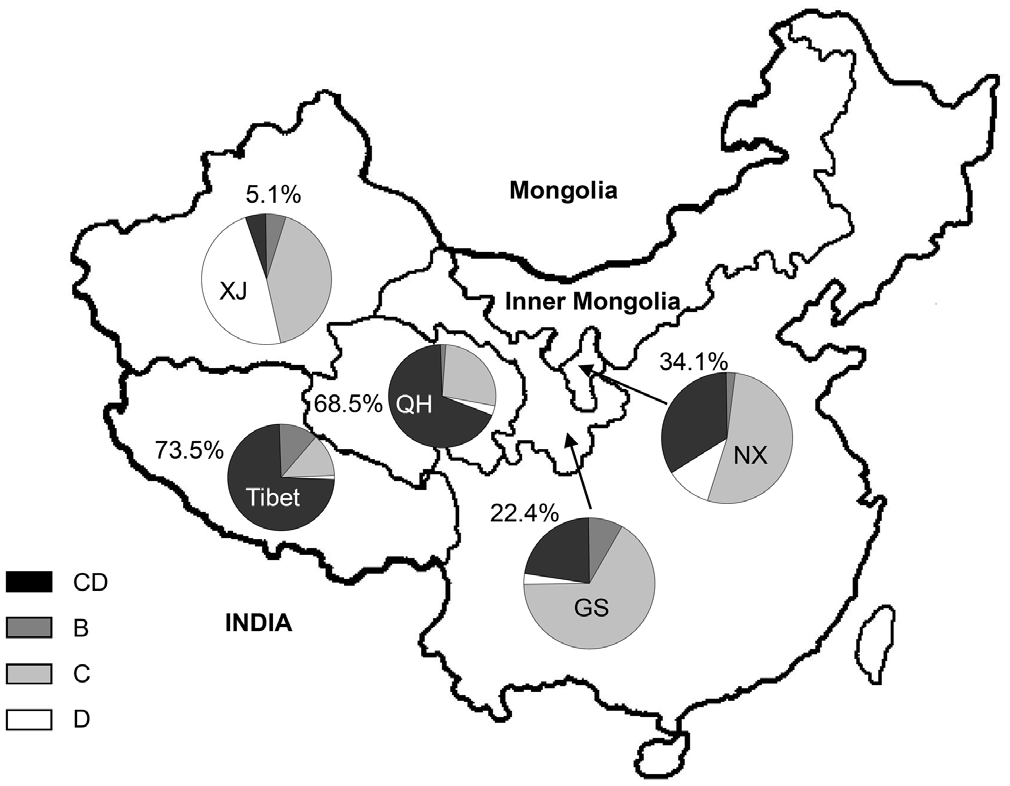
Distribution of HBV genotype by geographical location in western China. The proportion of the HBV C/D recombinants (‘CD1’ and ‘CD2’) in each region is shown. The complete names of regions mentioned in this figure are as follows: XJ = Xinjiang, QH = Qinghai, GS = Gansu, NX = Ningxia.

Geographically, the distribution of the C/D recombinant showed a gradient from east to west in western China (Fig. 1). In the eastern region (Ningxia and Gansu), HBV genotype C was the most common genotype with a much higher prevalence than the C/D recombinant (63.0% *vs.* 25.0%). In this region, 96.0% of the C/D recombinant belonged to the ‘CD1’ strain. In contrast, the C/D recombinant was the most prevalent HBV strain in the western region (Tibet), and had a much higher prevalence than genotype C (73.5% *vs.* 12.7%). In this region, HBV ‘CD2’ strain was slightly more prevalent than ‘CD1’ (53.3% *vs.* 46.7%). In Qinghai, which is located between Gansu and Tibet, 68.5% of patients were infected with the HBV C/D recombinant; among these, 92.0% of the strains were ‘CD1’. Thus, in patients from the east to the west region of western China, the prevalence of the HBV C/D recombinant showed an increasing tendency, whereas the prevalence of genotype C showed a decreasing tendency. Such divergent tendencies were also observed when the two types of the HBV C/D recombinant were considered: ‘CD1’ recombinant was remarkably prevalent in the east region, whereas ‘CD2’ had a higher prevalence in the west region.

Ethnically, the HBV C/D recombinant had a substantially higher prevalence in native aboriginal Tibetan patients than in local Han patients. An increasing prevalence of the C/D recombinant in native aboriginal patients was also observed from east to west in this region: in the eastern area, the prevalences were 34.1% and 64.5% in Ningxia and Gansu native Hui patients, respectively, whereas in the western area, the prevalences were 80.4% and 90.1% in Qinghai and Tibet native Tibetan patients, respectively. Notably, in Uygur patients of Xinjiang province, the prevalence of the HBV C/D recombinant (5.1%) was much lower than that of genotype D (48.7%) and C (41.0%).

### Clinical and virological differences between the C/D recombinant and subgenotype C2

**Figure 2:**
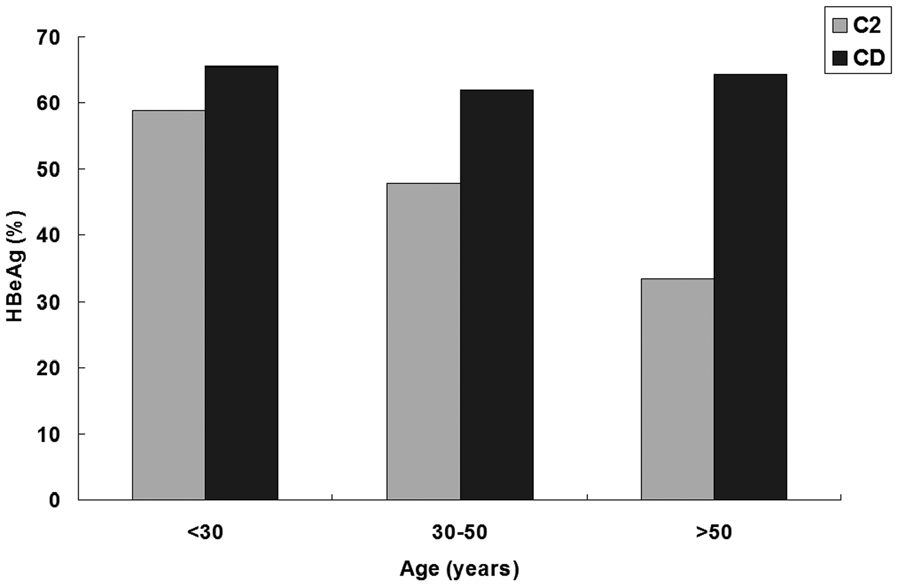
HBeAg positivity in patients of HBV/CD1 or HBV/C2. Patients were stratified by age into three groups, younger than 30 years (<30), 30–50 years and older than 50 years (>50). The prevalence of HBeAg decreased with age in patients of HBV/C2 (58.8% in <30 years; 48.0% in 30–50 years; 33.3% in >50 years), whereas almost no change was observed in patients of HBV/CD1 (21 of 32, 65.6% in <30 years; 39 of 63, 61.9% in 30–50 years; 9 of 14, 64.3% in >50 years). The rate of HBeAg positivity did not reach the statistical significance in all three age groups between HBV/CD1 and HBV/C2 (*P* = 0.758, 0.244 and 0.336 in <30, 30–50 and >50 years respectively).

Clinical data and prevalence of pre-core and core promoter mutations in patients infected with HBV/C2 and HBV/CD1, respectively are shown in Table 2. The level of serum total bilirubin (TBIL) in patients with HBV/C2 was significantly higher than that in patients with HBV/CD1. In the 48 subgenotype C2 infections, 32 were Han and 16 were Tibetan. In the 109 ‘CD1’ infections, 20 were Han and 89 were Tibetan. When the TBIL levels were compared between the 52 Han and the 105 Tibetan, no significant difference was found (27±34 vs. 19±32, *P* = 0.139) suggesting that the different TBIL levels between individuals with ‘CD1’ and C2 infection were not confounded by ethnic differences. The prevalence of hepatitis B e antigen (HBeAg) was higher in patients with HBV/CD1 (63.3%) than that in patients with HBV/C2 (50.0%), but the difference did not reach statistical significance (*P* = 0.118). When patients were stratified by age into three groups, the prevalence of HBeAg decreased with age in patients with HBV/C2 (10 of 17, 58.8% in <30 years; 12 of 25, 48.0% in 30–50 years; 2 of 6, 33.3% in >50 years), whereas kept at an almost constant high prevalence in patients with HBV/CD1 (21 of 32, 65.6% in <30 years; 39 of 63, 61.9% in 30–50 years; 9 of 14, 64.3% in >50 years). But the differences were not significant between patients with HBV/C2 and HBV/CD1 in all three stratified age groups patients (*P* = 0.758, 0.244 and 0.336 in <30, 30–50 and >50 years, respectively) (Fig. 2). The pre-core stop mutation (A1896) occurred significantly less often in patients with HBV/CD1 than in patients with HBV/C2 (4.6% vs. 18.8%, *P* = 0.004) though all the patients had T1858. Whereas the double mutations in the core promoter (T1762/A1764) seemed to occur more frequently in patients with HBV/CD1 than in patients with HBV/C2 though the difference did not reach statistical significance (32.1% vs. 22.9%, *P* = 0.244).

**Table 2:** Clinical and virological differences of HBV/CD1 and HBV/C2.

	C2 (n = 48)	CD1 (n = 109)	*P* value
Age (yrs)	34.7±12.9	34.9±11.7	0.939
Sex (n, Male/Female)	29/19	63/46	0.759
HBeAg (n, positive/negative)	24/24	69/40	0.118
Liver function indicators			
ALT (U/L)	119.1±229.7	102.6±200.0	0.649
AST (U/L)	94.7±144.2	95.7±300.2	0.983
TBIL (µmol/L)	31.4±49.6	17.4±21.5	0.015
Viral mutations (%)			
T1653	2 (4.2)	7 (6.4)	0.723
V1753	2 (4.2)	12 (11.0)	0.23
T1762/A1764	11 (22.9)	35 (32.1)	0.244
A1896	9 (18.8)	5 (4.6)	0.004

**NOTE.** Data are given as mean±SD or no. (%) of patients. ALT, alanine aminotransferase; AST, aspartate aminotransferase; TBIL, total bilirubin.

### Phylogenetic analysis of mosaic and backbone sequences of the HBV C/D recombinant

**Figure 3:**
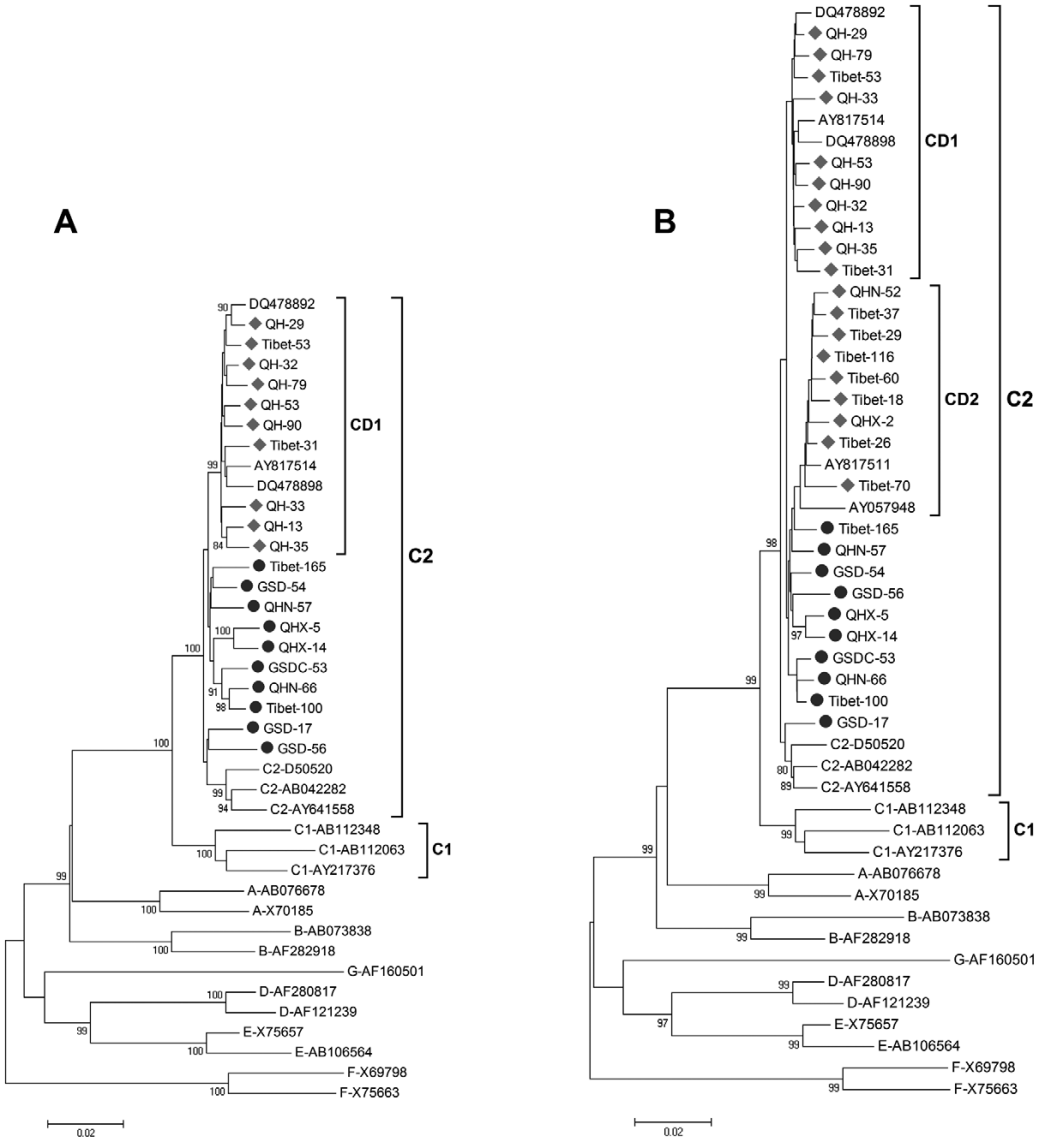
Phylogenetic analysis of backbone sequences of the HBV C/D recombinant compared with reference strains representing genotypes A–G. Accession numbers and sample numbers are shown on each branch, and are indicated on the left by the HBV genotype or subgenotype. Bootstrap values are shown along each main branch. Scale bars indicate the nucleotide divergence. Isolates determined in this study are marked. A, Phylogenetic tree based on nt 800–3215. B, Phylogenetic tree based on nt 1500–3215.

When the C/D recombinant isolates sequenced in the present study were compared to those from other studies [16], [17], [23] using SimPlot and phylogenetic analyses, they were found to have similar recombination breakpoints (data not shown). To determine whether the C fragment of the ‘CD1’ (nt 800-3215) and the ‘CD2’ (nt 1500-3215) recombinants originated from subgenotype C2. Two phylogenetic trees were constructed based on nt 800-3215 (Fig. 3A) and nt 1500-3215 (Fig. 3B) respectively. Fig. 3A includes 10 ‘CD1’, 10 subgenotype C2 strains isolated from local patients in this study, 3 ‘CD1’ isolates reported previously, and 17 reference sequences retrieved from GenBank representing HBV genotypes A–G. Fig. 3B includes another 11 ‘CD2’ strains isolated from local patients in this study and reported previously. The phylogenetic trees show that the HBV ‘CD1’ isolates were grouped separately in one cluster within subgenotype C2, although the bootstrap values were relatively low (45% in Fig. 3A and 53% in Fig. 3B), reflecting the difference between the backbone sequence of the ‘CD1’ recombinant and that of the local subgenotype C2 strain. The average distance between the ‘CD1’ and subgenotype C2 on the backbone sequence (nt 800-3215) was 2.15%. The average distance between the ‘CD2’ and C2 (nt 1500-3215) was 1.74%. The estimated mean substitution rate in HBV was 4.2×10^−5^ nucleotide substitutions/site/year. Applying this rate to the phylogenetic analysis, we estimated that the origin of ‘CD1’ may have occurred 520 years ago and ‘CD2’ may have occurred 410 years ago.

**Figure 4:**
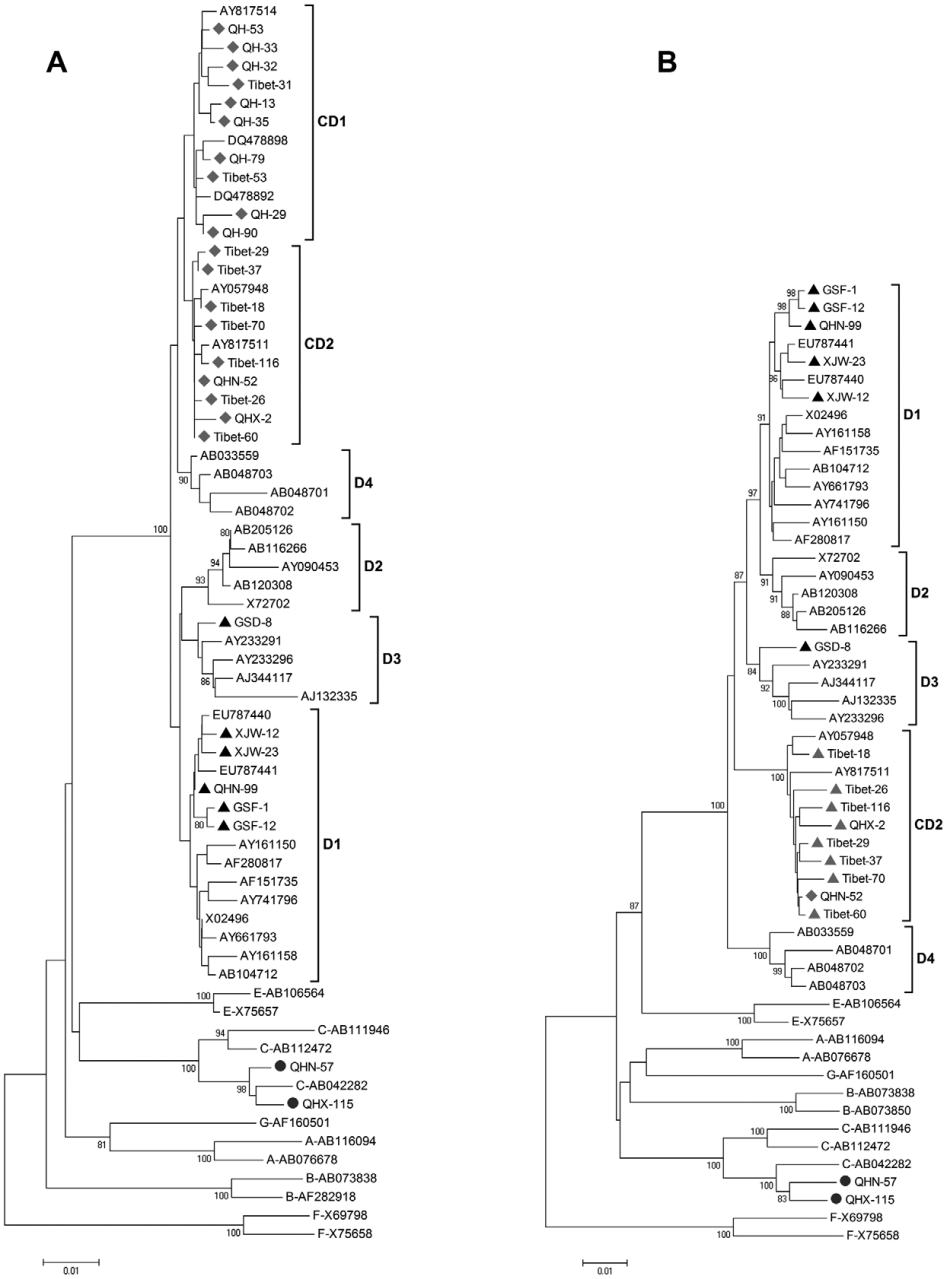
Phylogenetic analysis of mosaic sequences of the HBV C/D recombinant compared with reference strains representing genotypes A–G. Accession numbers and sample numbers are shown on each branch, and are indicated on the left by the HBV genotype or subgenotype. Bootstrap values are shown along each main branch. Scale bars indicate the nucleotide divergence. Isolates determined in this study are marked. A, Phylogenetic tree based on nt 10-799. B, Phylogenetic tree based on nt 10-1499.

To determine whether the D fragment of the ‘CD1’ (nt 10-799) and the ‘CD2’ (nt 10-1499) recombinants originated from genotype D. Two phylogenetic trees were constructed based on nt 10-799 (Fig. 4A) and nt 10-1499 (Fig. 4B) respectively. Fig. 4A includes 10 ‘CD1’, 9 ‘CD2’, 6 genotype D, 2 subgenotype C2 isolates from this study, and 40 reference sequences retrieved from GenBank representing HBV genotypes A–G. The phylogenetic trees show that the ‘CD1’ and the ‘CD2’ isolates clustered on another branch within genotype D, independently from the D1–D4 subgenotypes. The newly isolated genotype D strains from local patients were grouped in the D1 or D3 subgenotype. The average distance between the ‘CD1’ and genotype D on the mosaic sequence (nt 10-799) was 1.82%. The estimated divergence time of ‘CD1’ from genotype D is about 430 years. The average distance between the ‘CD2’ and genotype D on the mosaic sequence (nt 10-1499) was 3.49%. So the estimated divergence time of ‘CD2’ from genotype D is about 830 years.

## Discussion

Accumulating data have revealed the frequent existence of mosaic HBV genomes, which are generally considered to be the result of recombination between two different genotype strains. In western China, two types of genotype C/D recombinant have been identified [16], [17], [23]. In the present study, a large-scale survey on the geographical and ethnic distribution of the HBV C/D recombinant in western China reinforce the results of our previous reports based on smaller samples [23]. By analyzing a large cohort of 624 patients with chronic HBV infection from five provinces of western China, we found that the two types of C/D recombinant account for 51.9% of the patients, suggesting that the HBV C/D recombinants prevail in this region.

Our results show that the ‘CD1’ recombinant has a higher prevalence than the ‘CD2’ strain in western China, but that its distribution follows a gradient from east to west, such that the ‘CD1’ strain has a remarkably higher prevalence than the ‘CD2’ in the eastern part of western China. In Mongolia and Inner Mongolia, which are located in the north of Gansu and Ningxia provinces, genotype D is the most prevalent HBV strain, and ‘CD1’ recombinant was observed with a very low prevalence [24], [25], [26]. In contrast, we found that ‘CD2’ exists at a slight higher prevalence than ‘CD1’ in Tibet, contradicting a previous prediction that all C/D hybrids were ‘CD2’ recombinant in this region [16]. Interestingly, the further west, the more ‘CD2’ recombinant can be observed. Across the Himalayas, genotype D is the most prevalent HBV strain, followed by genotypes A and C in India and Nepal [27], [28], [29]. Our results demonstrate that the HBV C/D recombinant is restricted to a specific region in western China, mainly on the Qinghai-Tibet Plateau, which rises about 4000 meters above sea level. Around this region, genotype D is the predominant HBV strain in the west, north and south, whereas genotype C is the most prevalent HBV strain in the east [30], [31], [32], [33], [34].

The geographic origin of the HBV C/D recombinant remains largely unknown because of the shortage of evidence. In the light of the findings from this study we speculate on the possible geographic origin of HBV C/D recombinant. Of the five provinces in this study, Gansu and Xinjiang were on the Silk Road, a well-traveled transcontinental trade route that linked Europe (mainly genotype D) in the West with China (mainly genotype C) in the East. If the HBV C/D recombinant is the results of co-infection by genotype C and D followed by recombination, Gansu and Xinjiang should have a much higher prevalence of the C/D recombinant than Tibet and Qinghai. Contrary to this, our investigation shows that the HBV C/D recombinant is the predominant HBV strain in Tibet and Qinghai. The Qinghai-Tibet Plateau is a mountainous area with a series of huge mountain ranges, the Qilian, Kunlun, Tanggula, Gangdisi, and Himalayas, that run through from east to west. Because of the particularly high altitude, extreme environmental conditions, and the special religious traditions of this area, it is difficult for people from surrounding areas to enter the region. This physical and cultural isolation also prevents interbreeding and social contact of the Tibetan ethnic population with outside people. This may explain why the C/D recombinants once introduced and flourished there.

It is well known that HBV genotypes correlate well with ethnicity and geography, but the mechanism is still unclear. Study from hepatitis C virus has shown that adaptation to multiple host Human leukocyte antigen (HLA) alleles is an important cause of viral mutation and evolution, and thus divergence [35]. Previous investigations have shown the great polymorphism of HLA alleles among the Tibetan, Uygur, and Han populations in western China [36], [37]. In particular, although the Tibetan and northern Han Chinese populations shared many similar HLA alleles, they also had distinct frequencies of many HLA alleles and haplotypes. However, some allelic distributions in the Uygur population were more similar to those among Caucasians. These observations are coincidentally in agreement with the present investigation on HBV genotype distributions among these three ethnic populations, with genotype D having the highest prevalence among Uygur, the C/D recombinant being the highest among Tibetans, and genotype C being the highest among Han Chinese. It is still uncertain whether the high prevalence of the C/D recombinant in Tibetan population is the viral adaptation to the specific HLA alleles of Tibetan.

Traditionally, HBV recombination is presumed to be the consequence of genetic material exchange of two HBV strains after one patient has been co-infected with two different HBV genotypes. Different HBV genotype strains co-infection has been widely described [34], [38], [39], [40], but intergenotype recombination events have been rarely reported in co-infected patients [7], [11], [13]. A more recent study indicated that recombination in HBV is not as extensive as previously assumed by using an alternative software STRUCTURE [41]. For intergenotype recombinant, the mosaic sequences should be derived from the parental strains. When we phylogenetically analyzed the backbone sequences of ‘CD1’ and ‘CD2’ recombinants with the corresponding sequences of local subgenotype C2, we found that ‘CD1’ recombinants were grouped separately, instead of clustering among local C2 isolates within subgenotype C2 (Fig. 3). When we phylogenetically analyzed the mosaic fragments of ‘CD1’ and ‘CD2’ with the corresponding fragment of genotype D, we also observed that the C/D recombinants did not cluster with subtypes D1–D4, but rather formed separate clusters of its own (Fig. 4). Likewise, these findings suggest that the C/D recombinants (‘CD1’ and ‘CD2’) could have evolved after the recombination events and this has taken place over a long period of time. The ‘CD1’ and ‘CD2’ may have evolved over 500 years and 800 years, respectively.

Many studies have shown the association of HBV genotype/subgenotype and specific mutations with clinical outcome of HBV, though the role of HBV genotypes in response to antiviral therapy is still uncertain [42], [43], [44]. Studies from Asia populations suggested that genotype C was associated with an increased risk of hepatocellular carcinoma, lower rate of spontaneous HBeAg seroconversion and higher rate of core promoter double mutations (T1762/A1764) compared with genotype B [45], [46], [47]. A recent study from Alaska showed that HBeAg seroconversion occurred decades later in patients infected with HBV genotype C than in those infected with genotypes A, B, D and F, suggesting that genotype C may be responsible for most perinatal transmission [48]. In the present study, the prevalence of HBeAg in the carriers of HBV/CD1 was comparable with that in the carriers of HBV/C2, suggesting that patients with HBV/CD1 had the similar HBeAg duration to patients with HBV/C2. The T1762/A1764 and A1896 mutations are the most common HBeAg-negative variants that reduce or abolish HBeAg production. These two types of mutation pattern may be preferentially selected by different genotype HBV strain in developing HBeAg-negative infection [49]. Here we observed a significant lower tendency to develop A1896 (4.6% vs. 18.8%, *P* = 0.004), but a slight higher tendency to develop T1762/A1764 (32.1% vs. 22.9%, *P* = 0.244) mutations in patients with HBV/CD1 than in patients with HBV/C2, suggesting that the T1762/A1764 mutations were more selected than the A1896 mutation by the HBV C/D recombinant. The lower level of TBIL in patients with HBV/CD1 than in patients with HBV/C2 suggested that patients with HBV/CD1 may have a lower risk of liver damage. But it remains hard to conclude that HBV/CD1 has a lower capacity of disease-inducing than HBV/C2.

In summary, our present investigation shows that the HBV C/D recombinant is specifically restricted to the Qinghai-Tibet Plateau. This region is geographically located between East and West Asia where genotype C and D are the most prevalent HBV strains, respectively. HBV/CD1 had a lower tendency to develop A1896 mutation than HBV/C2, but the prevalence of HBeAg was comparable. Further studies are still needed to investigate the clinical significance of the HBV C/D recombinant and its association with hepatocarcinogenesis.
